# How high: Quantity as a predictor of cannabis-related problems

**DOI:** 10.1186/1477-7517-5-20

**Published:** 2008-05-29

**Authors:** Nicole Walden, Mitch Earleywine

**Affiliations:** 1 Department of Psychology, University at Albany, State University of New York, 1400 Washington Ave., SS369, Albany, New York, 12222, USA

## Abstract

**Background:**

Research on cannabis use has emphasized frequency as a predictor of problems. Studies of other drugs reveal that frequency relates to psychological and physiological outcomes, but quantity also plays an important role. In the study of cannabis, quantity has been difficult to assess due to the wide range of products and means of consumption.

**Methods:**

The present study introduces three new measures of quantity, and examines their contribution to cannabis-related problems. Over 5,900 adults using cannabis once or more per month completed an internet survey that inquired about use, dependence, social problems and respiratory health. In addition to detailing their frequency of cannabis use, participants also reported three measures of quantity: number of quarter ounces consumed per month, usual intensity of intoxication, and maximum intensity of intoxication.

**Results:**

Frequency of use, monthly consumption, and levels of intoxication predicted respiratory symptoms, social problems and dependence. What is more, each measure of quantity accounted for significant variance in outcomes after controlling for the effects of frequency.

**Conclusion:**

These findings indicate that quantity is an important predictor of cannabis-related outcomes, and that the three quantity measures convey useful information about use.

## Background

Most research on cannabis emphasizes frequency of consumption. For example, three prominent surveys, the National Epidemiological Survey on Alcohol and Related Conditions [1], The Epidemiologic Catchment Area [2], and the National Comorbidity Survey [3] inquire about the number of joints smoked per day, and how often participants used in the past twelve months. Research on other drugs reveals that frequency serves as an important predictor of problems, but quantity also plays an important role [4-6]. Similarly, tobacco research has determined that the impact of cigarette smoking on respiratory health is related to the quantity of tobacco smoked [7,8]. Thus, whether addressing the psychological or the physiological outcomes of cannabis use, there is impetus to consider quantity.

Quantity of cannabis used is an important predictor of psychosocial outcomes. In a study conducted by Stephens and colleagues [9] cannabis users meeting the criteria for dependence typically reported consuming nearly one half ounce of cannabis per week, and smoking approximately three joints daily. Thus, in this research population, a profile of heavy use accompanied dependence. Other studies indicate that frequency alone does not overwhelmingly account for dependence. For example, the 2003 National Survey on Drug Use and Health [10] found over 60% of daily cannabis users to be non-dependent. Such variance in dependence among even daily users suggests that other factors, such as quantity, play a role in dependence.

For those who smoke cannabis, quantity also contributes to respiratory symptoms. Inhaling cannabis smoke exposes the lungs to harmful gaseous and particulate matter [11,12] and is associated with respiratory impairment [13]. Daily cannabis smoking predicts respiratory symptoms comparable to those resulting from cigarette smoke, such as coughing, increased phlegm and shortness of breath [14]. Although the risks for lung cancer are markedly higher in cigarette smokers because of nicotine's tumor-enhancing effects and THC's tumor-inhibiting ones, the acute and longitudinal effects of cannabis smoke on respiratory functioning are dose dependent in a way that suggests that the smoke increases respiratory irritation [11,15]. In light of such findings on both respiratory health and psychosocial functioning, it is apparent that quantity plays an important role in determining the consequences of cannabis use.

While quantity of use is an important factor to consider, it is rarely measured. For example, of forty-one articles identified through the PsychInfo literature database that address cannabis use and psychosocial problems, only two include a measure of quantity [16,17]. In an effort to contend with "uncertainties about THC content [18]," researchers have often defined heavy use as "daily or near daily use [18]." Addressing quantity more directly, Chen and colleagues [16] and Grant and Pickering [17] measured quantity in terms of the number of joints smoked per day or per smoking occasion. These approaches have been helpful, and research stands to benefit from incorporating other direct and indirect measures of quantity.

The assessment of quantity is important for understanding outcomes of cannabis use, but the design of a meaningful measure is not without its challenges. Tobacco use may be relatively easy to quantify due to cigarette manufacturing standards, but there is no standardized cannabis product. Alcohol researchers have devised a "standard drink" [19] to measure alcohol consumption comparably between different beverage types. However, as joint size varies, THC content varies, and there are many different products and means of delivery, a "standard drink" measure may not apply to cannabis. Nevertheless, an estimate of amount consumed could still help predict problems. Additionally, as an indirect measure of quantity, an index of intoxication might also help predict problems.

The present study introduces new measures of quantity and examines the relation between quantity of cannabis consumption and problems. As a direct measure of physical quantity consumed, we inquired about the number of quarter ounces used per month. Cannabis users may not be able to report the precise dosage of individual joints, but are likely to be familiar with the rate at which they consume quarter ounces, as this unit of weight typically corresponds with product purchase [20,21]. In addition to this direct measure, we also measured quantity indirectly by asking about intensity of intoxication. Psychoactive effects are dose-related [22-25] and therefore intoxication level conveys information about quantity. We asked individuals to report both their usual and maximum levels of intoxication, as each may relate differentially to problems. With these direct and indirect measures, we were able to examine the independent contributions of frequency and quantity to cannabis-related outcomes.

Consistent with previous cannabis research [11,26-28], we hypothesized that frequency would predict respiratory symptoms, social problems, and dependence. We further hypothesized that quantity would predict respiratory symptoms, social problems, and dependence even after the effects of frequency were taken into account. To examine the independent contribution of each predictor, as well as the effects of quantity after controlling for frequency, hierarchical multiple regression analyses were conducted for each outcome.

## Methods

### Participants

Data were collected by means of an internet survey. In order to access a sample of regular cannabis users, participants were recruited from among the constituency of several organizations promoting drug law reform. The researchers requested The Drug Policy Alliance, The Marijuana Policy Project, and The National Organization for the Reform of Marijuana Laws to notify their membership of the survey through e-mail. Participants were asked to forward the survey information to others, and were entered into a drawing for a cash prize.

Data from 5,987 participants who reported using marijuana at least once per month was used for the analyses. These individuals included 3,884 men and 2,103 women ranging in age from 18 to 88 years (*M *= 31.24, *SD *= 12.33), with an average educational attainment between some years of college and an associate's degree. Participants were 89% Caucasian, 5% mixed race, 1% African/Caribbean descent, 1% Asian, 1% Latino, 1% Native American, and 2% chose not to specify ethnicity. Approximately 97% of participants were from the United States, 1% were from the regions of Oceania, Western and Northern Europe, and the remaining 2% were from a large variety of countries around the world, not representing any particular region.

The analysis examining respiratory symptoms included only those participants who did not have cystic fibrosis or asthma, and who had never used other inhalant drugs, in order to reduce the contribution of other factors related to respiratory health. This analysis was also limited to individuals whose primary method of cannabis consumption involved smoking. Therefore, those who reported consuming cannabis primarily by eating were excluded. As a result, data from a subsample of 2,647 participants was available for the analysis predicting respiratory symptoms. These participants were demographically equivalent to the larger sample.

### Procedure

#### Survey

The local Institutional Review Board approved the survey and informed consent procedure. Participants were informed of the nature of the study, and were instructed that survey items would inquire about their drug use and quality of life. Informed consent was obtained at the beginning of the survey.

#### Measures

Demographic variables of sex and age were significantly correlated with the outcomes of interest and were therefore included in the analyses as covariates.

#### Frequency

Participants reported how many days per month they used cannabis.

#### Quantity

As a direct measure of quantity, participants were asked to report the number of quarter ounces they used per month, on average.

#### Intensity of intoxication

Usual and maximum levels of intoxication were assessed to indirectly measure quantity of cannabis consumption. Participants reported the level of high they usually experienced ("Approximately how 'high,' 'buzzed,' 'stoned,' or intoxicated do you usually get when you use marijuana?"), as well as the maximum level of high experienced in the past 90 days ("What's the highest you've been in the past 90 days?"). To maximize consistency between individuals' ratings, response options were provided along a six-point scale describing a range of highs (1 = 'light buzz', 6 = 'very high') [25].

#### Respiratory symptoms

Six questions that have been used in previous work on cannabis smokers [12] inquired about respiratory symptoms: Do you have a cough? Do you cough up phlegm in the morning? Are you troubled by shortness of breath when hurrying on the level ground or walking up a slight hill? Do you have to walk slower than most people your own age on the level ground because of breathlessness? Does your chest sound wheezy or whistling other than from colds? Do you wake up at night with tightness in your chest? Participants responded 'yes' or 'no' and symptoms were summed to create a composite variable.

#### Social problems associated with cannabis use

Nineteen items from the Marijuana Problems Scale [29] assessed the experience of social problems related to cannabis use in the past 90 days. Items included such problems as getting into trouble at work, getting into fights, or losing friends because of cannabis use. Responses were made on a six-point scale of severity, anchored at 0 (no problem) and 5 (serious problem).

#### Dependence

Dependence symptoms were assessed using a seven-item measure based on the DSM-IV-TR criteria for substance dependence [16,30]. In each item, participants were asked to indicate whether or not they had experienced a particular dependence symptom within the past 12 months.

#### Cigarette smoking

Cigarette smoke contributes to similar respiratory symptoms as cannabis smoke [14,31]. Therefore, two measures of cigarette smoking were included as covariates in the analysis predicting respiratory symptoms. In one item, participants indicated if they had ever smoked cigarettes. In a second item, participants reported the average number of cigarettes they currently smoked per day.

## Results

Participants' cannabis use ranged in frequency from 1 to 31 days per month over the past year (*M *= 22.62, *SD *= 10.01), with an average monthly consumption of 3/8 of an ounce (*SD *= 0.65, range = 0 to 3/4 ounce). A mean of 0.58 respiratory symptoms was reported (*SD *= 0.96), with responses ranging from no symptoms to all six symptoms. Participants reported experiencing an average of 1.37 dependence symptoms (*SD *= 1.42, range of 0 to 7) over the past year, and an average of 4.02 cannabis-related social problems (*SD *= 1.57, range of 0 to 19) in the past 90 days. The distributions of responses for cannabis-related problems, quarter ounces consumed per month and dependence symptoms were skewed. Subsequently, these variables were transformed to normalize the distribution of responses. Usual and maximum intoxication levels ranged from the lowest to the highest response option. The average level of usual intoxication was near the midpoint (*M *= 3.70, *SD *= 1.26), and the average level of maximum intoxication approached the high end of the scale (*M *= 5.09, *SD *= 1.29).

Hierarchical multiple regression analyses were conducted to examine the degree to which frequency and quantity of cannabis use predicted respiratory symptoms, social problems and dependence symptoms. Separate regression analyses were conducted for each outcome, with participants' age, sex, frequency of cannabis use, quantity of consumption, usual high, and maximum high entered as predictors in each analysis. Additionally, the analysis predicting respiratory symptoms included measures of cigarette smoking as covariates. Significance levels for the second and third analyses were corrected to prevent inflated probability of type I error [32].

In all analyses, age and sex were included as covariates in step 1. In the analysis predicting respiratory symptoms, number of cigarettes currently smoked per day, and whether participants had ever smoked cigarettes were also included in step 1 as covariates. Next, in all analyses, frequency of use was entered in step 2. Quantity predictors were then entered individually, to examine their unique contribution to outcomes. Quantity of monthly consumption was entered in the third step of regression, usual intoxication level in the fourth, and maximum intoxication level in the fifth and final step. Inter-item correlations are provided in Table 1. Results of the regression analyses indicate that the three quantity variables contributed significantly to the prediction of respiratory symptoms, social problems and dependence, over and above frequency of use.

**Table 1:** Intercorrelations Between Independent Variables (N = 5987)

Variables	1	2	3	4	5	6	7	8
1. Respondent age	---							
2. Respondent sex	-0.005	---						
3. Ever smoked cigarettes	0.115**	0.092**	---					
4. Number of cigarettes per day	0.254**	0.057**	0.688**	---				
5. Frequency of use (days per month)	0.047**	-0.021	0.159**	0.180**	---			
6. Quarter ounces consumed per month	-0.006	-0.014	0.159**	0.238**	0.600**	---		
7. Usual intoxication intensity	-0.310**	-0.082**	-0.048**	-0.078**	0.052**	0.136**	---	
8. Maximum intoxication intensity	-0.424**	-0.087**	-0.021	-0.086**	0.195**	0.168**	0.629**	---

*Note*. **p < 0.01

### Respiratory Symptoms

Altogether, the predictors for respiratory symptoms explained just over 14 % of variance in this outcome. Frequency of use, quarter ounces per month, and usual intoxication level were significantly related to respiratory symptoms, in order of increasing associative strength (see Table 2). The number of respiratory symptoms reported increased the more frequently cannabis was smoked, the greater the quantity smoked, and the greater the usual level of intoxication. Participants' maximum level of intoxication was found to be negatively related to respiratory symptoms. This finding was unexpected, and further replication would be necessary before this result could be interpreted. After controlling for the effects of frequency, each of the three quantity measures accounted for unique and significant variance in respiratory symptoms. With the exception of maximum intoxication, all effects were in the expected direction.

**Table 2:** Summary of Regression Analysis for Variables Predicting Respiratory Symptoms (N = 1868)

Variable	*B*	*SE B*	β	*R*^2^	*R*^2^*-Change*
Step 1				0.124	0.107***
Respondent sex	0.219	0.047	0.102***		
Respondent age	-0.006	0.002	-0.063*		
Ever smoked cigarettes	0.115	0.064	0.057		
Cigarettes smoked per day	0.028	0.003	0.268***		
Step 2				0.134	0.010***
Frequency of use (days per month)	0.194	0.088	0.062*		
Step 3				0.140	0.005**
Quarter ounces per month	0.297	0.094	0.087**		
Step 4				0.142	0.002*
Usual intoxication intensity	0.073	0.023	0.091**		
Step 5				0.144	0.002*
Maximum intoxication intensity	-0.059	0.025	-0.074**		
*F *(8,1868) = 39.267, *p *< 0.001.					

*Note*. *p < 0.05, **p < 0.01, ***p < 0.001

### Social Problems

The set of predictors in this analysis accounted for approximately 12% of variance in participants' report of problems associated with cannabis use. As shown in Table 3, frequency of consumption and each of the three quantity measures were positively related to the experience of cannabis-related social problems. Those who used cannabis more often reported a significantly greater number of social problems associated with cannabis use. After controlling for frequency of use, the effect of quarter ounces consumed per month was in the predicted direction, but did not reach conventional significance levels (*R*^2 ^*change *= .001, *p *= .054). Both usual and maximum intoxication levels did significantly predict problems, with more intense 'highs' corresponding with the experience of more social problems.

**Table 3:** Summary of Regression Analysis for Variables Predicting Social Problems (N = 5828)

Variable	*B*	*SE B*	β	*R*^2^	*R*^2^*-Change*
Step 1				0.084	0.084***
Respondent sex	-0.134	0.037	-0.045***		
Respondent age	-0.028	0.002	-0.246***		
Step 2				0.113	0.029***
Frequency of use (days per month)	0.663	0.073	0.142***		
Step 3				0.113	0.001^*α*^
Quarter ounces per month	0.078	0.073	0.017		
Step 4				0.122	0.008***
Usual intoxication intensity	0.075	0.018	0.067***		
Step 5				0.124	0.002**
Maximum intoxication intensity	0.065	0.019	0.058**		
*F *(6,5827) = 136.881, *p *< 0.001.					

*Note*. **p < 0.01, ***p < 0.001 ^*α *^*p *= 0.054

### Dependence

As shown in Table 4, the independent variables accounted for approximately 21% of variance in the number of dependence symptoms reported. Frequency of use and all three measures of quantity were significantly related to the endorsement of dependence symptoms. More frequent use predicted the endorsement of more dependence symptoms. What is more, each of the three quantity measures accounted for a significant portion of variance in dependence symptoms, over and above frequency. Greater monthly consumption of quarter ounces was associated with a higher number of dependence symptoms, after controlling for the contribution of frequency. Both usual and maximum intoxication levels were positively related to the number of dependence symptoms reported, after controlling for frequency and quantity of quarter ounces consumed.

**Table 4:** Summary of Regression Analysis for Variables Predicting Dependence (N = 5446)

Variable	*B*	*SE B*	β	*R*^2^	*R*^2^*-Change*
Step 1				0.097	0.097***
Respondent sex	-0.022	0.006	-0.046***		
Respondent age	-0.005	0.000	-0.265***		
Step 2				0.179	0.082***
Frequency of use (days per month)	0.140	0.012	0.183***		
Step 3				0.194	0.016***
Quarter ounces per month	0.110	0.012	0.143***		
Step 4				0.202	0.008***
Usual intoxication intensity	0.011	0.003	0.058***		
Step 5				0.205	0.003***
Maximum intoxication intensity	0.013	0.003	0.070***		
*F *(6,5445) = 233.836, *p *< 0.001					

*Note*. ***p < 0.001

## Summary

The results demonstrate that quantity is an important indicator of cannabis-related outcomes. The first hypothesis was supported, as frequency was found to be a significant predictor of respiratory symptoms, social problems and dependence. The second hypothesis was also supported. Quantity of cannabis use predicted respiratory symptoms, social problems and dependence, after controlling for the effects of frequency. Each of the three quantity measures was positively related to dependence symptoms. Two of the quantity measures, quantity of monthly consumption and level of usual intoxication, were positively associated with respiratory symptoms. When examining social problems, more intense usual and maximum intoxication levels significantly predicted a higher number of problems. In addition to predicting outcomes over and above frequency, each of the three quantity measures related significantly to outcomes when controlling for the effects of each other. These findings indicate that monthly consumption, usual and maximum intoxication levels not only predict cannabis-related outcomes, but that each measure conveys distinct information.

## Discussion

The present study provides evidence that quantity of cannabis use is an important predictor of both psychosocial and physiological outcomes. Variability in products, dosage and delivery has posed a challenge to the quantification of cannabis use, and past research has typically focused on frequency. To explore the contribution of quantity to cannabis-related outcomes, we introduced three new measures of quantity and examined their relation to respiratory symptoms, social problems and dependence.

Quantity was measured directly by inquiring about the number of quarter ounces participants consumed per month. As an indirect measure of quantity, we also asked participants to report their usual and maximum levels of intoxication. Consistent with the extant literature, frequency of use predicted social problems and dependence, as well as respiratory symptoms among cannabis smokers. After controlling for the effects of frequency, quantity of cannabis use also predicted respiratory symptoms among smokers, social problems and dependence. Each of the three quantity measures accounted for significant and unique variance in outcomes, indicating that monthly consumption, maximum and typical intoxication levels each convey distinct information related to outcomes of cannabis use.

Although the observed effects of frequency were notably larger than those of quantity, even the relatively small effects of quantity bear considerable consequence in an epidemiological context. With over 40% of people in the United States trying cannabis once or more [33], and a prevalence of problems in regular users up to 12% [34], this relatively small effect could relate to a large number of people. When addressing such important outcomes as respiratory function, social problems and dependence, each amount of variance that can be accounted for is beneficial. Given the popularity of cannabis, there is all the more impetus to understand the dimensions of use that are relevant to wellbeing.

There are limitations to the interpretation of results due to sampling methodology. Participants were recruited from organizations that advocate drug policy reform, and could have been hesitant to report negative experiences associated with cannabis use. The occurrence of problems might therefore be underrepresented in the data. However, a full range of responses was observed for each outcome, suggesting that participants were likely to have been forthcoming. Previous research also indicates that substance use may be reported at least as candidly in Internet surveys as in paper-based surveys, if not more so [35,36]. Therefore, the use of web-based survey methodology may have increased the likelihood of honest self report.

The extent that the present sample represents the larger population of cannabis users is unclear. Internet survey methods enable data to be collected from a large, geographically diverse group of individuals, but respondents may differ from the population at large. In the present study, participants were recruited via the Internet, and the majority of the sample consisted of Caucasian Americans with some amount of college education. For these reasons, the characteristics of these participants may not generalize to others of different demographic backgrounds, those with lower educational attainment, or individuals who may not have access to the internet. An astute and anonymous reviewer emphasized that the role of medical use could prove particularly important in these results. We failed to assess the role of medicinal use in this study. Medical users might require relatively large quantities of cannabis while experiencing few negative side effects like those assessed here. A responsible medical user could consume large amounts without numerous symptoms of social problems, dependence, or respiratory irritation. Thus, the current results may be overestimates of the potential impact of quantity on problems in medical users. More importantly, the potential positive effects of cannabis overall and in relation to quantity do not appear in these data. Medical users may report greater symptom relief as quantities increase, or an optimal dose that is neither too high nor too low. Recreational users may also find that the effects that they appreciate most will vary with quantity, perhaps with an optimal dose that is neither too high nor too low. The results on respiratory effects also suggest that using smaller amounts of higher quality cannabis could enhance the safety of the plant. Examining the implications of quantity in more diverse populations would require different sampling and survey methods.

Clearly, quantity of cannabis consumed can be an important predictor of problems. Further research can employ the three new measures of quantity to help clarify its role in outcomes. These single-item indicators each reveal unique aspects of the amount consumed and contribute to the prediction of negative consequences. Continued efforts to examine quantity can have important implications for prevention and treatment of cannabis-related problems. As another astute and anonymous reviewer mentioned, these data support the idea that efforts to increase the safety of cannabis can emphasize decreasing amount as well as frequency of consumption. Despite the challenges to quantification, the present findings suggest that perhaps researchers and clinicians should not only ask people how often they smoke; they should also ask how much they consume and how high they get.

## Competing interests

ME is affiliated with organizations devoted to changing cannabis laws.

## Authors' contributions

NW managed data, performed analyses, and drafted the manuscript, ME contributed to study design, coordination of data collection, and manuscript revisions.
